# Repeated Influenza Vaccination Boosts and Maintains H1N1pdm09 Neuraminidase Antibody Titers

**DOI:** 10.3389/fimmu.2021.748264

**Published:** 2021-10-14

**Authors:** Lena Hansen, Fan Zhou, Håkon Amdam, Mai-Chi Trieu, Rebecca Jane Cox

**Affiliations:** ^1^ Influenza Centre, Department of Clinical Science, University of Bergen, Bergen, Norway; ^2^ Department of Microbiology, Haukeland University Hospital, Bergen, Norway

**Keywords:** influenza, neuraminidase, neuraminidase inhibition, neuraminidase inhibition (NAI) titer, repeated vaccination, pre-existing immunity, pandemic vaccination

## Abstract

Antibodies to influenza surface protein neuraminidase (NA) have been found to reduce disease severity and may be an independent correlate of protection. Despite this, current influenza vaccines have no regulatory requirements for the quality or quantity of the NA antigen and are not optimized for induction of NA-specific antibodies. Here we investigate the induction and durability of NA-specific antibody titers after pandemic AS03-adjuvanted monovalent H1N1 vaccination and subsequent annual vaccination in health care workers in a five-year longitudinal study. NA-specific antibodies were measured by endpoint ELISA and functional antibodies measured by enzyme-linked lectin assay (ELLA) and plaque reduction naturalisation assay. We found robust induction of NA inhibition (NAI) titers with a 53% seroconversion rate (>4-fold) after pandemic vaccination in 2009. Furthermore, the endpoint and NAI geometric mean titers persisted above pre-vaccination levels up to five years after vaccination in HCWs that only received the pandemic vaccine, which demonstrates considerable durability. Vaccination with non-adjuvanted trivalent influenza vaccines (TIV) in subsequent influenza seasons 2010/2011 – 2013/2014 further boosted NA-specific antibody responses. We found that each subsequent vaccination increased durable endpoint titers and contributed to maintaining the durability of functional antibody titers. Although the trivalent influenza vaccines boosted NA-specific antibodies, the magnitude of fold-increase at day 21 declined with repeated vaccination, particularly for functional antibody titers. High levels of pre-existing antibodies were associated with lower fold-induction in repeatedly vaccinated HCWs. In summary, our results show that durable NA-specific antibody responses can be induced by an adjuvanted influenza vaccine, which can be maintained and further boosted by TIVs. Although NA-specific antibody responses are boosted by annual influenza vaccines, high pre-existing titers may negatively affect the magnitude of fold-increase in repeatedly vaccinated individuals. Our results support continued development and standardization of the NA antigen to supplement current influenza vaccines and reduce the burden of morbidity and mortality.

## Introduction

Influenza is an acute respiratory disease that is annually estimated to cause 3 – 5 million cases of severe illness and 290 000 – 650 000 deaths worldwide (1, 2). Influenza vaccines are currently the most effective method of prevention of influenza infection. Hemagglutinin (HA) is the major surface glycoprotein on the virus that mediates viral entry by binding to sialic acid receptors on the surface of host cells. Antibodies that target the HA globular head and block binding to sialic acids are considered the classical mediators of protection against influenza infection. These antibodies are measured by hemagglutination inhibition (HI) assay and the HI titer has been the gold standard for measuring vaccine immunogenicity for many years. Thus, current seasonal influenza vaccines are optimized for induction of HA-specific antibodies. Each vaccine dose is standardized by the HA content, however, there are no concentration requirements for the other vaccine components, such as neuraminidase. Neuraminidase (NA) is the second major surface glycoprotein. It is a sialidase that cleaves terminal sialic acids and facilitates the release and spread of newly formed viruses from host cells (3). HI antibodies tend to be strain-specific and have reduced cross-reactivity with new and drifted influenza strains due to the high mutation rate of the HA head region. Antigenic drift and shift may occur independently for HA and NA proteins and NA is a potential target for more broadly protective vaccines. Early studies established that NA is immunogenic and that NA-specific antibodies reduce disease in humans (4, 5). More recent studies have found that antibodies with NA inhibition (NAI) activity correlate with reduced viral shedding and clinical disease, and may be a possible correlate of protection (6, 7). Despite NA being an antigenic target for induction of protective antibody responses, the quantity and quality of NA is not regulated in current influenza vaccines. Consequently, the amount and stability of the NA antigen has been found to vary between influenza vaccines and key epitopes targeted by human monoclonal antibodies are poorly displayed (8, 9). Studies have reported variable seroconversion rates for NA-specific antibody responses after vaccination with inactivated influenza vaccines, ranging between 23 – 64% (7, 10, 11).

Annual vaccination is recommended due to antigenic drift of influenza viruses and waning of antibody titers. However, there is growing evidence showing that repeated influenza vaccination can lead to a diminished B cell response (12) and that high pre-existing antibody titers can reduce boosting of antibody titers after vaccination (13). The impact of repeated vaccination has mainly been studied in the context of HA-specific antibody responses (12, 13). Thus, there is limited data on whether repeated vaccination and pre-existing titers impact the induction of NA-specific antibody responses after vaccination.

This study aimed to investigate the induction and durability of NA-specific antibodies with AS03-adjuvanted pandemic H1N1pdm09 vaccination and determine the impact of subsequent annual vaccination with trivalent inactivated vaccines (TIV) in health care workers (HCWs). We found that AS03-adjuvanted monovalent H1N1pdm09 vaccination induced robust and durable NA-specific antibody responses. The antibody titers were further boosted after immunization with TIVs, however, we found that the magnitude of the functional NA antibody fold-increase declined with repeated vaccination.

## Materials and Methods

### Study Design and Blood Sampling

Healthy HCWs (n=50) were vaccinated between October 2009 and March 2010 at Haukeland University Hospital, Norway with the AS03-adjuvanted pandemic H1N1pdm09 split virus vaccine (3.75 μg hemagglutinin A/California/7/2009 (H1N1) (Pandemrix, GlaxoSmithKline-GSK, Belgium). Written informed consent was obtained before inclusion in the study. Further informed consent was obtained for the 4-year extension between 2010/2011–2013/2014 where vaccination was with the trivalent seasonal inactivated influenza vaccine [TIV; either subunit (Influvac, Abbott Laboratories) or split-virion (Vaxigrip, Sanofi Pasteur)] containing 15 μg hemagglutinin per strain. Throughout the study, the A/H1N1 strain remained the same [A/California/07/2009 (H1N1)], however the A/H3N2 and B viruses changed between seasons. Demographic and clinical information including working department were collected. The study was approved by the regional ethics committee (REKVest-2012/1772) and the Norwegian Medicines Agency (Clinical trials.gov NCT01003288) (14).

Blood samples were collected pre-vaccination (D0), 21 days (D21), 3, 6, and 12 months (3M, 6M, 12M, respectively) after vaccination. Annual influenza vaccination is recommended, but not mandatory for HCWs in Norway. The HCWs were divided into two groups, repeated and single group, based on their vaccination status in influenza seasons 2010/2011 – 2013/2014. The single group did not receive any TIVs during the study. The repeated group was vaccinated with two or three TIVs in the four seasons following the 2009 pandemic. An overview of the number of vaccinations and the intervals that these were given for the repeated group can be found in **Supplemental Table 2**. The same sampling schedule was followed after all vaccinations. The 12M timepoint was collected from all HCW irrespective of vaccination and used as D0 for HCWs in the repeated group for each season. Blood samples collected before vaccination were considered as day 0 whenever HCWs had not been vaccinated during the previous season, which more accurately reflected the true baseline titers. HCWs in the single group (n=24) were only vaccinated in 2009 but provided yearly blood samples at the start of each influenza season, i.e. 24, 36, 48 and 60 months after H1N1pdm09 vaccination. The samples collected from the single group after the 2009 season were labelled as 12M for each subsequent season as seen in **Figure 2**. All serum samples were heat inactivated at 56°C for one hour before use in serological assays.

### ELISA

Flat bottom 96-well plates (Invitrogen) were coated with recombinant N1 NA A/California/07/2009 (H1N1) (Cal09) produced in a baculovirus expression system as previously described (15). N1 NA Cal09 (100 µl/well) diluted in PBS (1 µg/ml) was added and incubated overnight at 4°C. The plates were washed six times with PBS-T (PBS with 0.05% Tween 20) and blocked with 200 µl of blocking solution [PBS with 0.1% Tween-20 (Sigma), 1% BSA (Sigma), 5% milk (Marvel)] and incubated for 1 hour at 37°C. Sera were 4-fold serially diluted from 1:100 in blocking solution and 100 µl of diluted serum was added per well in duplicates and incubated at 37°C for 1 hour. Following incubation, the plates were washed six times with PBS-T and 100 µl horseradish peroxidase (HRP)-conjugated mouse anti-human IgG (BD Biosciences) diluted in blocking buffer (1:4000) was added per well and incubated at 37°C for 1 hour. The plates were washed six times with PBS-T and the secondary antibody signal was developed by adding 100 µl per well of 3,3′,5,5′-Tetramethylbenzidine (TMB) substrate (BD Biosciences). The reaction was stopped after 10 min by adding 100 µl of 1M HCl (Sigma). Absorbance was measured at 450 and 620 nm with a microplate reader (Bio-Tek). Background measured at 620 nm was subtracted from the absorbance measured at 450 nm. The endpoint titer was determined using a sigmoidal dose response curve in GraphPad Prism 9.

### ELLA

Inhibition of NA enzyme activity was determined using enzyme-linked lectin assay (ELLA) using an influenza reassortant H7N1 virus (NIBSC, UK) with an irrelevant HA from A/Equine/ Prague/56 (H7N7) and NA from A/California/07/09 (H1N1), matching the vaccine strain. ELLA was performed according to Couzens et al. (16). Briefly, 96-well plates were coated with 100 µl/well of fetuin (25 µg/ml) (Sigma) diluted in PBS and incubated at 4°C for a minimum of 18 hours. The plates were washed three times with PBS-T. Sera were 5-fold serially diluted from 1:50 in sample diluent (PBS with 0.9 mM CaCl_2_ and 0.5 mM MgCl_2_ (Life Technologies), 1% BSA (Sigma), 0.5% Tween-20) and 50 µl was added per well in duplicates. The virus was diluted in sample buffer at a concentration equivalent to 90% of the maximum signal and 50 µl was added per well. The plates were incubated at 37°C for 18 hours. After incubation, the plates were washed six times with PBS-T and 100 µl of HRP-conjugated peanut agglutinin (1 mg/ml) (Sigma) diluted in conjugate diluent (PBS with 0.9 mM CaCl_2_ and 0.5 mM MgCl_2_, 1% BSA) was added to per well and incubated in the dark at room temperature for 2 hours. The plates were washed three times with PBS-T. 100 µl of o-Phenylenediamine dihydrochloride (OPD) substrate (Sigma) in phosphate-citrate buffer (50 mM) (Sigma) was added to each well and incubated in the dark for 10 min. The reaction was stopped by adding 100 µl 1N sulfuric acid (Sigma). The absorbance was measured at 490 nm. 50% inhibitory concentration was calculated for each serum sample using a sigmoidal dose response curve in GraphPad Prism 9 and considered as the neuraminidase inhibition (NAI) titer.

### Plaque Reduction Neutralization Assay

A 96-well microplate plaque reduction neutralization assay was used to measure the capacity of sera to inhibit viral replication *in vitro*. The reassortant H7N1 virus used in ELLA was used for this assay. The plaque reduction neutralization assay was performed according to Matrosovich et al. (17, 18). MDCK SIAT1 cells were seeded (2x10^4^ per well) and incubated overnight at 37°C. The virus was diluted to a concentration that would generate 100 plaques per well. Sera were diluted 1:20 and 1:100 and mixed with the virus, and incubated at 37°C for one hour. This inoculum was added in quadruplets and incubated at 37°C for 40 min. A low-viscosity Avicel overlay (FMC BioPolymer) was added to each well and the plates were incubated with the inoculum-overlay mixture for 24 hours. The plaques were visualized by immunostaining of nucleoprotein and the plaques were counted using ELISpot counter (AID). The number of plaques in the control wells was used to determine 50% inhibition and the highest reciprocal dilution giving 50% reduction in plaque formation was defined as plaque reduction neutralizing titer (PRNT_50_ titer). The NA inhibitor oseltamivir (Roche) was used as a positive control to confirm that the assay could detect reduction in plaque forming units (PFU) as a result of NA inhibition in a dose-dependent manner (**Supplementary Figure 1**). Human serum depleted for IgA, IgM, and IgG (Sigma) was used as negative control.

### Statistical Analysis

Endpoint, NAI and PRNT_50_ titers were log-transformed and analyzed by linear mixed effects model with adjustments for demographic factors and multiple comparisons by Sidak correction. Demographic factors used for adjustments included age, sex, influenza vaccination prior to 2009 and working department (**Table 1**). The linear-mixed effects model analyses were performed in IBM SPSS Statistics version 26 and was the statistical test used unless otherwise stated. Analyzes of statistical difference between the single and repeated group was done by non-parametric Kruskal-Wallis test in GraphPad Prism 9. Correlation coefficients were calculated by non-parametric Spearman correlation in GraphPad Prism 9. Statistical significance was defined as P<0.05 for all tests.

**Table 1:** Demographics of the study participants.

Demographics	All HCWs	Single group	Repeated group
Number of participants	50	24	26
Male (%)	9 (18)	4 (17)	5 (19)
Female (%)	41 (82)	20 (83)	21 (81)
Median age (range)	39 (22 – 63)	38 (26 – 59)	43 (22 – 63)
Seasonal vaccination before 2009 (%)	32 (65.3)	11 (47.8)	21 (80.8)
Working department(non-clinical, clinical, infectious)	23, 21, 6	13, 9, 2	10, 12, 4

HCW healthcare workers (HCWs). The single group had only pandemic vaccination in 2009. The repeated group were vaccinated with pandemic vaccine in 2009 and trivalent influenza vaccine in two or three seasons after 2009.

## Results

In this study we investigated NA-specific antibody responses after pandemic vaccination and subsequent annual influenza vaccination. Fifty HCWs were included in the study, which consisted of 9 men and 41 women. These numbers reflect the gender distribution in the Norwegian healthcare system. The median age at the start of the study was 38 years old (range 22 – 63). The majority of the HCWs (65%) had been vaccinated with TIVs before 2009 (**Table 1**).

### Robust and Durable NA-Specific Antibody Responses After 2009 Pandemic Vaccination

**Figure 1:**
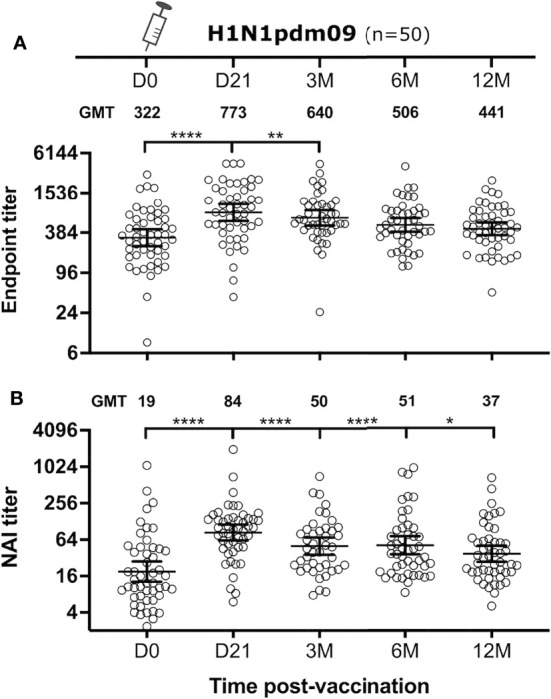
NA-specific antibody responses after H1N1pdm09 vaccination. Health care workers (n = 50) were vaccinated with AS03-adjuvanted H1N1pdm09 vaccine. The NA-specific antibody response was measured before and 21 days, 3, 6 and 12 months after vaccination (D0, D21, 3M, 6M, 12M, respectively). Endpoint titers were measured by ELISA **(A)** and NA inhibition (NAI) titers were measured by ELLA **(B)**. Each subject is shown as one symbol with geometric mean and 95% confidence intervals. A linear mixed effects model was used for statistical analyses between pre- and post-vaccination titers with adjustments for demographic factors. *P ≤ 0.05, **P ≤ 0.01, ****P ≤ 0.0001.

One objective of this study was to investigate the induction of NA-specific antibody responses after AS03-adjuvanted monovalent pandemic vaccine (H1N1pdm09) in healthy adults. All HCWs in the study were vaccinated with the H1N1pdm09 vaccine in 2009 and blood samples were collected before and 21 days, 3, 6 and 12 months after vaccination. Endpoint titers were determined by ELISA against recombinant N1 NA Cal09 (**Figure 1A**). NA-specific endpoint titers were detected in all HCWs pre-vaccination with a geometric mean titer (GMT) of 322. The endpoint titers were significantly boosted to a GMT of 773, a geometric fold rise (GMFR) of 2.4, at 21 days post-vaccination (P<0.0001). The endpoint titers gradually waned during the following 12 months, although titers were significantly higher than pre-vaccination levels up to the 3-month time point (P=0.004).

The capacity of the antibody response to inhibit NA enzyme activity was measured by ELLA. A reassortant H7N1 virus, with an irrelevant H7 HA from A/Equine/Prague/02/56 and N1 NA from Cal09, was used to avoid interference from HA-specific antibodies. The H1N1pdm09 vaccine met the European Committee for Medicinal Products for Human Use (CHMP) criteria for immunogenicity set for HI titers (**Supplementary Table 1**). These criteria were used to assess induction of NAI titers. Pre-vaccination NAI titers were detected in all HCWs, albeit with a modest NAI GMT of 19. H1N1pdm09 vaccination significantly increased NAI GMT at 21 days post-vaccination to 84 (P<0.0001), a 4.4-fold increase from pre-vaccination levels (**Figure 1B**). The seroconversion rate for NAI titers was 53% (32/49). Similarly to endpoint titers, the NAI titers gradually waned but remained significantly higher than the pre-vaccination level up to 12 months after vaccination (P=0.014).

**Figure 2:**
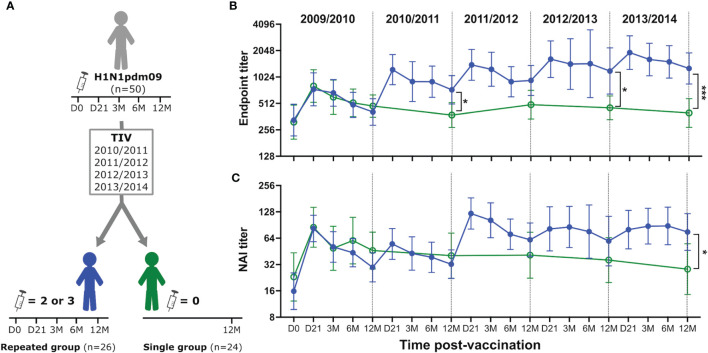
H1N1pdm09 vaccination induced durable NA-specific antibody responses boosted by trivalent influenza vaccines. Study design **(A)**. Health care workers were divided into two groups based on their vaccination status after H1N1pdm09 vaccination in 2009. The repeated group (blue, filled circles) received two or three trivalent influenza vaccines (TIV) during seasons 2010/2011-2013/2014. Blood samples were collected 21 days (D21), and 3, 6, 12 months (3M, 6M, 12M, respectively) after vaccination. The single group (green, open circles) chose not to be further vaccinated but provided yearly blood samples prior to the start of each season (12M). NA-specific antibody responses were measured by ELISA **(B)** and ELLA **(C)** during each influenza season from 2009/2010 to 2013/2014. Data are shown as geometric mean with 95% confidence intervals. Analyzes of statistical difference between the single and repeated group was done by non-parametric Kruskal-Wallis test in GraphPad Prism 9. *P ≤ 0.05, ***P ≤ 0.001.

Approximately half of the HCWs (26/50) chose to receive two or three TIVs during the four-year follow-up study after the 2009 pandemic (repeated group). The remaining 24 HCWs chose not to be further vaccinated (single group) (**Figure 2A**). The repeated group had higher endpoint and NAI titers than the single group, especially during the influenza season (defined as November – April) (**Figures 2B, C**). Annual blood samples were collected prior to the start of each season from the single group, which allowed us to investigate the durability of antibody responses induced by the H1N1pdm09 vaccine. We found that three HCWs in the single group seroconverted during the study (HI titer >4-fold increase), probably due to infection. Samples collected after seroconversion were excluded to ensure that the durability was only measured from pandemic vaccination. Endpoint and NAI titers were maintained at low stable levels above baseline in the single group throughout the study (**Figures 2B, C**). The GMFR was 1.38- and 1.33-fold above pre-vaccination levels for endpoint and NAI titers, respectively, five years after H1N1pdm09 vaccination. This demonstrated that a single dose of AS03-adjuvanted H1N1pdm09 vaccine induced durable antibody responses that persisted for several years after vaccination, suggesting that long-lived plasma cells were generated.

### Trivalent Influenza Vaccines Boost NA-Specific Antibody Responses

**Figure 3:**
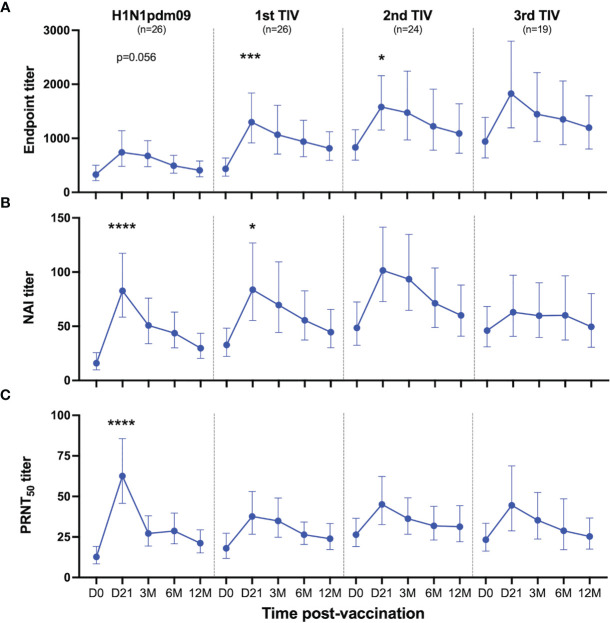
Antibody responses to NA induced by H1N1pdm09 and TIV vaccination. Health care workers in the repeated group received two or three trivalent influenza vaccines (TIV) in the four subsequent seasons after the 2009 pandemic during the five-year study. Blood samples were collected pre-season (D0) and 21 days, 3, 6 and 12 months (D21, 3M, 6M, 12M, respectively) after each vaccination. Antibody responses were measured by ELISA **(A)**, ELLA **(B)** and plaque reduction neutralization assay **(C)** after vaccination with H1N1pdm09 and the first, second or third TIV. All vaccinated HCWs in the repeated group is included in this figure regardless of their vaccination intervals. Data are shown as geometric mean with 95% confidence intervals. A linear mixed effects model with adjustments for demographic factors was used to determine statistical difference between antibody titers measured on day 0 and day 21 for each vaccination. *P ≤ 0.05, ***P ≤ 0.001, ****P ≤ 0.0001.

HCWs in the repeated group received two or three TIVs during the study, resulting in variation in vaccination intervals. The majority of the HCWs received three TIVs (81%), whereas the remaining HCWs received two TIVs (19%) (**Supplementary Table 2**). We observed that HCWs that had delayed their first TIV until the 2011/2012 season (6/26) had higher NAI titer than the HCWs that received their second TIV that season. Therefore, HCWs in the repeated group were grouped based on number of vaccines, rather than season, in order to study the effect of each vaccine and the impact of repeated vaccination. The quantity of NA-specific antibodies were measured by ELISA and functional antibodies were measured by ELLA and a plaque reduction neutralisation assay in all HCWs in the repeated group. The three assays demonstrated that NA-specific antibody responses were boosted after TIV vaccination (**Figure 3**). These antibody titers gradually decreased but persisted above baseline levels throughout the influenza season. We found that endpoint titers were significantly boosted after the first (P=0.001) and second TIV (P=0.04) (**Figure 3A**), whereas the NAI titers were only significantly boosted on day 21 after the first TIV (P=0.018) (**Figure 3B**). Although the third TIV boosted endpoint and NAI titers, neither were significant. Plaque reduction neutralisation assay was used to further assess the functionality of the NA-specific antibody response *in vitro* using the same reassortant H7N1 virus with N1 NA Cal09. This assay measures inhibition of the viral replication cycle and NA-specific inhibition of plaque formation was confirmed using oseltamivir (**Supplementary Figure 1**). We found a significant increase in antibody titer that resulted in 50% reduction in plaque formation (PRNT_50_ titer) 21 days after H1N1pdm09 (P<0.0001) (**Figure 3C**). Vaccination with TIVs boosted PRNT_50_ titers, although not significant for any of the three TIVs. The PRNT_50_ titers confirmed our findings in the ELLA and demonstrated that the vaccine-induced NA-specific antibodies were capable of inhibiting enzyme activity and viral replication *in vitro*. Collectively, our results show that seasonal TIV vaccination readily boosts NA-specific antibody responses following priming with AS03-adjuvanted H1N1pdm09 vaccination.

### Repeated Vaccination Increases Durable Endpoint Titers and Maintains Durability of Functional Antibody Titers

Durable antibody titers were measured 12 months after vaccination. The H1N1 component remained the same in all vaccines used during this study, which allowed us to investigate the impact of repeated vaccination with the same antigen. We compared endpoint and NAI titres in the repeated and single group at the end of the 5-year study to assess the impact of TIVs on the durability of antibody titers. Only HCWs in the repeated group that had been vaccinated with TIV in the final season of the study were used for comparison using samples collected 12 months post-vaccination. 14/18 HCWs in the repeated group had received three TIVs and 4/18 had received two TIVs in that season (**Supplementary Table 2**). We found that the repeated group had 3.2-fold higher endpoint titers (P=0.0002) (**Figure 2A**) and 2.7-fold higher NAI titers (P=0.01) (**Figure 2B**) compared to the single group 5 years after H1N1pdm09 vaccination. This demonstrated that repeated vaccination with TIVs after AS03-adjuvanted H1N1pdm09 vaccination contributed to maintenance and further increase of the durable NA-specific antibody responses.

**Figure 4:**
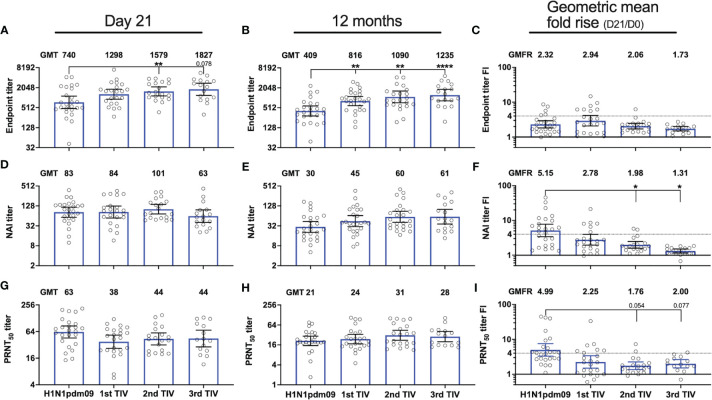
Repeated influenza vaccination boosted durable NA-specific antibody titers but with diminished fold-induction. NA-specific antibody responses were measured 21 days and 12 months after vaccination with AS03-adjuvanted monovalent H1N1pdm09 vaccine and three trivalent influenza vaccines (TIVs) in the four subsequent years after the 2009 pandemic. Antibody titers were measured by ELISA **(A, B)**, ELLA **(D, E)** and plaque reduction neutralization assay **(G, H)** in the repeated group. Geometric mean titers (GMT) are indicated for each vaccine on top of all graphs **(A, B, D, E, G, H)**. Fold-increase (FI) for each subject on day 21 (day 21/day 0) was calculated for the individual vaccines and the geometric mean fold rise (GMFR) for the whole group is indicated on top of each graph **(C, F, I)**. The dotted line represents the threshold set for seroconversion (>4-fold increase). Results are grouped by H1N1pdm09 vaccination and number of trivalent influenza vaccines (TIV) received. The data are presented as geometric mean with 95% confidence intervals. A linear mixed effects model with adjustments for demographic factors was used to determine statistical difference between antibody titers measured after vaccination with H1N1pdm09 and TIVs. *P ≤ 0.05, **P ≤ 0.01, ****P ≤ 0.0001.

Immunization with three TIVs gradually increased the magnitude of durable endpoint titers and collectively increased the GMT by 3-fold from titers measured 12 months after the H1N1pdm09 vaccination (**Figure 4B**). Durable antibody levels measured 12 months after the first (P=0.008), second (P=0.001) and third TIV (P<0.0001) were all significantly higher than the level measured after H1N1pdm09 vaccination (**Figure 4B**). This demonstrates that all TIVs contributed to further increase and maintenance of durable endpoint titers. The first TIV increased NAI titers 12 months after vaccination, which reached an antibody ceiling for the two subsequent TIVs (**Figure 4E**). No change was observed in PRNT_50_ titers after vaccination with TIVs (**Figure 4H**). Overall, our results indicate that the TIVs increased durable endpoint titers and maintained the durability of functional antibody titers.

### Repeated Vaccination Boosts Antibody Titers but With Reduced Fold-Increase

We analyzed the impact of repeated vaccination on endpoint titers, and functional NAI and PRNT_50_ titers measured at 21 days and 12 months after vaccination in the repeated group. The TIVs boosted endpoint titers measured on day 21 and the endpoint GMT increased gradually with each TIV (**Figure 4A**). The endpoint GMT was measured at 740 after H1N1pdm09 vaccination and was significantly higher after the second TIV when GMT increased to 1579 (P=0.039). Among the HCWs receiving the second TIV, 7/26 had not been vaccinated the year before and one HCW had not been vaccinated for two years (**Supplementary Table 2**). The endpoint GMT further increased to 1827 after the third TIV, which was the highest level measured during the study, although this was not significantly different from the endpoint GMTs measured after H1N1pdm09 or the other TIVs. The number of HCWs that received a third TIV was 19/26, however, two HCWs did not provide day 21 samples. Of these 19 HCWs, only 4 had not been vaccinated the season prior to the third TIV (**Supplementary Table 2**). We observed a different trend for the functional NAI titers on day 21. Although NAI titers were boosted by the TIVs, there were minimal differences in GMT on day 21 after the first, second and third TIV (**Figure 4D**). In fact, NAI GMT measured on day 21 was lowest after the third TIV. PRNT_50_ titers were also boosted after vaccination with TIVs and the highest GMT was observed 21 days after H1N1pdm09 vaccination, whereas TIVs induced lower but similar titers (**Figure 4G**). The level of PRNT_50_ titers measured 21 days after vaccination was not significantly different among the TIVs.

Although TIV vaccination boosted NA-specific antibody responses, the magnitude of the fold- increase on day 21 was not augmented by the number of vaccinations reflecting an antibody ceiling (**Figures 4C, F, I**). The GMFR for functional NAI and PRNT_50_ titers in the repeated group was highest after H1N1pdm09 vaccination and declined with each subsequent TIV (**Figures 4F, I**). The GMFR for NAI titers was significantly lower after the second (P=0.038) and third TIV (P=0.014) compared to H1N1pdm09 vaccination. A trend of decreasing GMFR for the PRNT_50_ titers was observed after the second (P=0.054) and third TIVs (P=0.077) although not significant compared to H1N1pdm09 vaccination. The seroconversion rate for NAI titers also declined with each TIV, where none of the HCWs had >4-fold increase after the third TIV. Seroconversion rates for the first and second TIV were 27 and 11%, respectively. Reduction in GMFR for endpoint titers was also observed with repeated vaccination but the effect was less pronounced than for functional antibody titers (**Figure 4C**).

**Figure 5:**
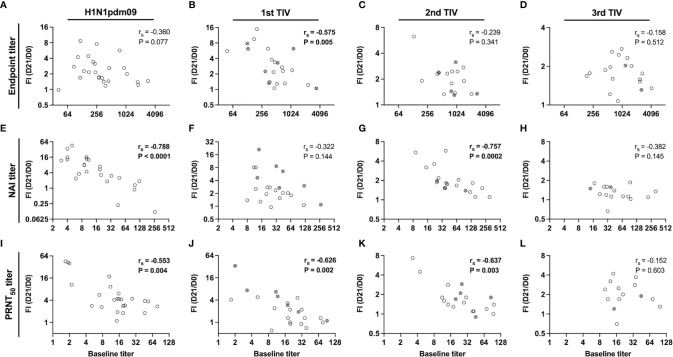
Relationship between pre-existing titers and fold-increase 21 days post-vaccination. Correlation between baseline titers and fold-increase (FI) of titers on day 21 post-vaccination (day 21/day 0) for endpoint **(A–D)**, NAI **(E–H)** and PRNT_50_ titers **(I–L)** in the repeated group. Health care workers (HCWs) were vaccinated with H1N1pdm09 and two or three trivalent influenza vaccines (TIVs) during influenza seasons 2009/2010 – 2013/2014. The antibody responses have been grouped by the vaccine number that were administered. All vaccinated HCWs in the repeated group is included in this figure regardless of their vaccination intervals. Open circles represent HCWs that were vaccinated the previous season and crossed circles represent HCWs that had chosen to not be vaccinated the previous season. The correlation coefficient (r_s_) was calculated by non-parametric Spearman correlation.

Pre-existing antibody titers to HA may have a negative effect on boosting of antibody titers after vaccination, however, it is unknown if this applies to NA-specific responses. Spearman correlation analysis was performed to investigate the relationship between baseline titers and fold-increase 21 days after vaccination (**Figure 5**). We found trends of inverse correlation, which was strongest for functional NAI (**Figures 5E–H**) and PRNT_50_ titers (**Figures 5I–L**). Significant inverse correlations were found for NAI titers after H1N1pdm09 and second TIV, and after H1N1pdm09 and the two first TIVs for PRNT_50_ titers. In contrast, a significant correlation was only found for endpoint titers after the first TIV (**Figures 5A–D**). Although the sample size was low, HCWs did not have a higher fold-increase when they had not been vaccinated during the previous year compared to those who had been vaccinated in the previous year (**Figure 5**). Overall, our results indicate that vaccination boosts and maintains NA-specific antibody titers but the magnitude of fold-increase at day 21 is reduced by repeated vaccination with the same vaccine strain over five years. The level of pre-existing antibody titers influenced the magnitude of fold-increase, particularly for functional antibody titers.

## Discussion

NA is required to be present in current influenza vaccines, however, there are no regulatory requirements for the amount or the quality of the antigen. Historically, NA content has not been regulated for vaccines due to a lack of standardized assays for measuring the NA concentration and also NA immunogenicity. Recent studies have emphasized the importance of NA-specific antibodies in protection against influenza disease and found that it may be an independent correlate of protection (6, 7). Here, we studied NA-specific antibody responses in HCWs after AS03-adjuvanted monovalent H1N1pdm09 vaccination in 2009 and annual vaccination with TIVs in the four subsequent influenza seasons. Our main finding is repeated influenza vaccination contributes to durable functional H1N1pdm09 NA-specific antibodies, although there is a reduced magnitude of fold-induction with increasing number of TIVs.

Early studies have shown that individuals with high pre-existing HAI titers have reduced boosting after re-vaccination (19, 20). Others have since reported that repeated vaccination and high pre-existing titers may reduce boosting of B cell responses and antibody titers after vaccination (12, 13, 21, 22). These studies have mostly focused on HA-specific antibody responses, however, lower antibody responses to NA after a second vaccination have been reported in individuals vaccinated in two consecutive years (23). Here we show that repeated vaccination has a similar effect on the NA-specific antibody response also after a third and fourth vaccination. We found that, although antibody titers were boosted after repeated vaccination, the magnitude of fold-induction declined, which was most prominent for functional NAI and PRNT_50_ titers. Additionally, the NAI and PRNT_50_ titer fold-increase on day 21 was inversely correlated with pre-existing titers. Others have hypothesized that boosting after repeated vaccination could be limited by pre-existing immunity and several mechanisms has been suggested based on mathematical modeling, which includes epitope masking model (24). This model proposes that pre-existing antibodies will bind and mask epitopes, blocking B cells that bind to the same or nearby epitopes, which results in limited B cell stimulation and expansion. Epitope masking may be a possible explanation for why the functional NA antibodies (measured by fold-increase for NAI and PRNT_50_ titers) peak after H1N1pdm09 vaccination but declines after subsequent TIVs, whereas this effect is less prominent for total NA-specific IgG binding antibodies measured by ELISA. Functional antibodies that are capable of conferring NAI activity bind directly or close to the enzyme active site, however, antigenic sites for human mAbs that do not have functional NAI activity have been described (25). Masking of NAI epitopes would still allow for stimulation of B cells reactive to other parts of the NA, which are readily measured by ELISA. Our observational study showed that repeated vaccination with the same strain induced durable NA-specific antibodies although it may reduce the magnitude of fold-increase, particularly functional antibodies capable of NAI. The persistence of durable antibody titers suggests that long-lived plasma cells are generated after adjuvanted H1N1pdm09 and subsequent non-adjuvanted TIVs. Further studies are warranted to understand the immunological mechanisms influencing this. This study was unique because the H1N1 vaccine component was the same for five consecutive influenza seasons. Antibody responses after vaccination with TIV during seasons 2006 – 2013 was found to be highest whenever one or more of the vaccine strains varied from year to year (21). More diversification of the H1N1 vaccine strain or addition of adjuvant could possibly have reduced the negative impact of repeated vaccination on antibody responses to NA and should be taken into consideration when choosing the vaccine strains for seasonal vaccines in the future.

A second objective of our study was to investigate the induction of NA-specific antibodies after AS03-adjuvanted monovalent H1N1pdm09 vaccination in healthy adults. This vaccine only contained 3.75 µg HA per dose due to dose-sparing during the 2009 pandemic, whereas TIVs are required to contain 15 µg HA. Quantification of the H1N1pdm09 vaccine composition by mass spectrometry revealed that one dose contained 21% HA and 6.9% NA, demonstrating that the amount of NA was even lower than that of HA per dose. Based on this estimation, the amount of NA would have been 1.23 µg per dose (26). Despite this, we found robust induction of NA-specific antibodies and a 53% seroconversion rate for NAI titers defined seroconversion as >4-fold increase. Other studies have found seroconversion rates ranging from 23 – 64%, however, the definition of seroconversion in these studies varied from 2 – 4-fold increase on day 21 from baseline (7, 10, 11). The seroconversion rate in our study is among the highest reported after influenza vaccination. This shows that robust and durable NA-specific antibody responses can be induced, even with low amounts of antigen when given with an appropriate adjuvant. Our results further support that standardization of the amount and stability of the NA antigen should be implemented for optimization of current influenza vaccines.

NA may undergo antigenic drift and shift independently of HA and NA immunity could provide protection in the event of mismatching of vaccine and circulating strains, and possibly against newly emerging strains. Broadly reactive NA antibodies have been described, demonstrating the breadth of immunity that could potentially be achieved through vaccination (8, 27). Standardizing the amount and supplementing current vaccines with NA have been proposed as a strategy for improving NA immunogenicity (28). A high dosage influenza vaccine containing eight times more NA activity than standard TIVs was found to induce higher levels of NAI antibodies compared to the standard TIV dosage in humans (29). Furthermore, computationally designed NA antigens tested in mice have shown that NA antigens can be designed for optimal cross reactivity (30). These strategies could possibly overcome the influence of pre-existing immunity and aid in the design of diverse antigens for optimal NA immunogenicity.

The current study has several limitations. HCWs may have been infected with influenza virus during the five-year study. This was more easily identified in the single group by increases of HI titers during a season, three HCWs were excluded from further analysis after seroconversion had occurred. However, this was more complicated for HCWs in the repeated group because it is not possible to distinguish increase in HI titer induced by vaccination *versus* infection. Furthermore, HCWs in the repeated group had different intervals of TIV vaccination and the sample size for the various regimens was low. However, we did not find that HCWs that had not been vaccinated for one or two years had higher antibody titer fold-increase compared to those who received TIVs in consecutive years. Only serological responses were measured in this study and therefore it is not known if and how the B cells are affected by repeated vaccination. Others have found inverse correlations between HA-specific pre-existing titers and the number of vaccine-induced antibody secreting cells (12, 21). Investigating this relationship for NA-specific humoral responses is important in future work as it could provide a better understanding of the interplay between pre-existing immunity and boosting, and its role in repeated influenza vaccination.

In conclusion, we found that AS03-adjvuanted pandemic vaccination boosted the NA-specific antibodies that persisted above pre-vaccination levels for 5 years. Repeated vaccination boosted NA-specific antibody titers, although with reduced the magnitude of fold-increase, particularly for functional antibodies. It is important to emphasize that vaccination is the best method of preventing influenza infection and annual vaccination remains beneficial. Our results support continued development and standardization of the NA antigen to supplement current influenza vaccines and reduce the burden of morbidity and mortality.

## Data Availability Statement

The raw data supporting the conclusions of this article will be made available by the authors, without undue reservation.

## Ethics Statement

The studies involving human participants were reviewed and approved by Regionale komiteer for medisinsk og helsefaglig forskningsetikk Vest. The patients/participants provided their written informed consent to participate in this study.

## Author Contributions

LH, FZ, and HA performed the experiments. LH and M-CT performed statistical analyses. LH and RJC wrote the manuscript. RJC and M-CT conceptualized and designed the study. All authors contributed to the article and approved the submitted version.

## Funding

LH was supported by Norwegian Research Council grant 271160. This study received intramural funding from the Influenza Centre at the University of Bergen and Haukeland University Hospital. The Influenza Centre is funded by the University of Bergen, Ministry of Health and Care Services, Helse Vest (F-11628), the Trond Mohn Foundation (TMS2020TMT05), the European Union (EU IMI115672 FLUCOP, H2020 874866 INCENTIVE, H2020 101037867 VACCELERATE, EU IMI101007799 Inno4Vac) and Nanomedicines Flunanoair (ERA-NETet EuroNanoMed2, JTC2016), and the Research Council of Norway GLOBVAC program (284930).

## Conflict of Interest

The authors declare that the research was conducted in the absence of any commercial or financial relationships that could be construed as a potential conflict of interest.

## Publisher’s Note

All claims expressed in this article are solely those of the authors and do not necessarily represent those of their affiliated organizations, or those of the publisher, the editors and the reviewers. Any product that may be evaluated in this article, or claim that may be made by its manufacturer, is not guaranteed or endorsed by the publisher.

## Supplementary Material

Supplementary Figure 1 NA inhibitor oseltamivir was used to verify that the plaque reduction neutralization assay was capable of measuring reduction of plaque forming units per well (PFU/well) as a result of NA inhibition in a dose-dependent manner.
