# Supplementary figures and images for: Repeated Influenza Vaccination Boosts and Maintains H1N1pdm09 Neuraminidase Antibody Titers
Source: Front Immunol. 2021 Oct 14;12:748264. doi: 10.3389/fimmu.2021.748264 (PMC8551669; doi:10.3389/fimmu.2021.748264)

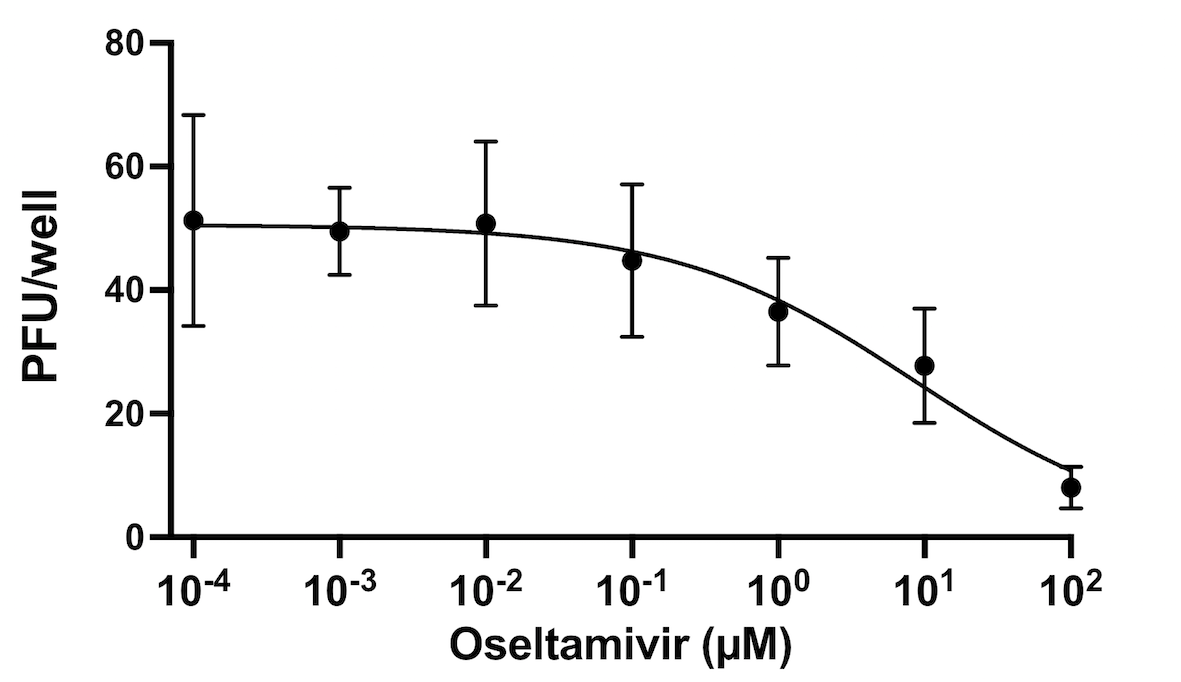

Supplement: Supplementary Figure 1 — NA inhibitor oseltamivir was used to verify that the plaque reduction neutralization assay was capable of measuring reduction of plaque forming units per well (PFU/well) as a result of NA inhibition in a dose-dependent manner. [file Image_1.tiff]
