# Supplementary material for: Repeated Influenza Vaccination Boosts and Maintains H1N1pdm09 Neuraminidase Antibody Titers
Source: Front Immunol. 2021 Oct 14;12:748264. doi: 10.3389/fimmu.2021.748264 (PMC8551669; doi:10.3389/fimmu.2021.748264)
Supplement: Supplementary file 2 [file Table_1.docx]

**Supplemental table 1.** CHMP immunological criteria for influenza vaccines

| **CHMP criteria for HI titer** | | **All HCWs (day 21)** |
| --- | --- | --- |
| Geometric mean fold rise | >2.5 | 49.4 |
| Seroconversion rate (>4-fold increase) | >40% | 92% |
| Seroprotection rate (HI titer >40) | >70% | 100% |

European Medicines Agency Committee for Medicinal product for human use (CHMP)
