# Supplementary material for: Repeated Influenza Vaccination Boosts and Maintains H1N1pdm09 Neuraminidase Antibody Titers
Source: Front Immunol. 2021 Oct 14;12:748264. doi: 10.3389/fimmu.2021.748264 (PMC8551669; doi:10.3389/fimmu.2021.748264)
Supplement: Supplementary file 3 [file Table_2.docx]

**Supplemental table 2. Overview of vaccination intervals and number of vaccinations in the repeated group**

| Vaccine intervals  2009-2010-2011-2012-2013 | HCWs  n (%) |
| --- | --- |
| 1-0-0-1-1 | 1 (4) |
| 1-0-1-0-1 | 2 (8) |
| 1-1-0-0-1 | 1 (4) |
| 1-1-1-0-0 | 1 (4) |
| 1-0-1-1-1 | 5 (19) |
| 1-1-0-1-1 | 5 (19) |
| 1-1-1-0-1 | 4 (15) |
| 1-1-1-1-0 | 7 (27) |

HCW healthcare workers (HCWs).
